# Towards a Central Role of *ISL1* in the Bladder Exstrophy–Epispadias Complex (BEEC): Computational Characterization of Genetic Variants and Structural Modelling

**DOI:** 10.3390/genes9120609

**Published:** 2018-12-05

**Authors:** Amit Sharma, Tikam Chand Dakal, Michael Ludwig, Holger Fröhlich, Riya Mathur, Heiko Reutter

**Affiliations:** 1 Department of Neurology, University Clinic Bonn, 53105, Bonn, Germany; Amit.Sharma@ukbonn.de; 2 Department of Ophthalmology, University Hospital Bonn, 53127 Bonn, Germany; 3 Department of Biotechnology, Mohanlal Sukhadia University Udaipur, 313001, Rajasthan, India; tikam260707@gmail.com; 4 Department of Clinical Chemistry and Clinical Pharmacology, University Hospital of Bonn, 53105 Bonn, Germany; mludwig@uni-bonn.de; 5 Bonn-Aachen International Center for IT, University of Bonn, 53115 Bonn, Germany; frohlich@bit.uni-bonn.de; 6 Department of Biosciences, Manipal University Jaipur, 303007 Jaipur, Rajasthan, India; knowme.riya@gmail.com; 7 Institute of Human Genetics, University Hospital of Bonn, 53127 Bonn, Germany; 8 Department of Genomics, Life & Brain Center, 53127 Bonn, Germany; 9 Department of Neonatology and Pediatric Intensive Care, Children’s Hospital, University of Bonn, 53113 Bonn, Germany

**Keywords:** *ISL1*, classic bladder exstrophy, STRING analysis

## Abstract

Genetic factors play a critical role in the development of human diseases. Recently, several molecular genetic studies have provided multiple lines of evidence for a critical role of genetic factors in the expression of human bladder exstrophy-epispadias complex (BEEC). At this point, *ISL1* (ISL LIM homeobox 1) has emerged as the major susceptibility gene for classic bladder exstrophy (CBE), in a multifactorial disease model. Here, GWAS (Genome wide association studies) discovery and replication studies, as well as the re-sequencing of *ISL1*, identified sequence variants (rs9291768, rs6874700, c.137C > G (p.Ala46Gly)) associated with CBE. Here, we aimed to determine the molecular and functional consequences of these sequence variants and estimate the dependence of ISL1 protein on other predicted candidates. We used: (i) computational analysis of conserved sequence motifs to perform an evolutionary conservation analysis, based on a Bayesian algorithm, and (ii) computational 3D structural modeling. Furthermore, we looked into long non-coding RNAs (lncRNAs) residing within the *ISL1* region, aiming to predict their targets. Our analysis suggests that the ISL1 protein specific N-terminal LIM domain (which harbors the variant c.137C > G), limits its transcriptional ability, and might interfere with ISL1-estrogen receptor α interactions. In conclusion, our analysis provides further useful insights about the *ISL1* gene, which is involved in the formation of the BEEC, and in the development of the urinary bladder.

## 1. Introduction

The bladder exstrophy–epispadias complex (BEEC) is the most severe of all human congenital anomalies of the kidney and urinary tract (CAKUT), and involves the abdominal wall, pelvis, all of the urinary tract, the genitalia, and occasionally the spine and anus [1]. Within the severity-spectrum of the BEEC, classic bladder exstrophy (CBE) represents the most common form, with an estimated birth-prevalence of about 1 in 37,000 live births having exstrophy–epispadias complex and bladder abnormalities [2].

Despite advances in surgical techniques, and improved understanding of the underlying anatomical defects, in later life many male and female patients experience chronic upper and lower urinary tract infections, sexual dysfunction, and urinary, or in the case of cloacal exstrophies, both urinary and fecal incontinence [1].

Recently, using genome-wide association methods in CBE patients of Central European background, we found an association with a region of approximately 220 kb on chromosome 5q11.1. This region harbors the *ISL1* (ISL LIM homeobox 1) gene, a master control gene expressed in pericloacal mesenchyme. Multiple markers in this region showed evidence for an association with CBE, including 84 markers with genome-wide significance [3]. In this study, the most significant marker (rs9291768) achieved a *P* value of 2.13 × 10^−12^. In a follow-up study with 268 CBE patients of Australian, British, German, Italian, Spanish, and Swedish origin; 1354 ethnically matched controls; and 92 CBE case-parent trios from North America; we were able to replicate this association. A meta-analysis of marker rs6874700 from our previous genome wide association study (GWAS) and our follow-up study, achieved a *P* value of 9.2 × 10^−19^. In a very recent re-sequencing study of *ISL1* in 125 BEEC patients of Swedish background, Arkani et al. (2018) detected 21 single nucleotide variants including a potentially novel missense variant, c.137C > G (p.Ala46Gly); substituting an amino acid, strictly conserved at its position as far down as Xenopus [4]. This variant was inherited from an unaffected mother. Using developmental biology models, we characterized the location of ISL1 activity in the forming urinary tract. Genetic lineage analysis of ISL1 expressing cells by the lineage tracer mouse model, showed ISL1-expressing cells in the urinary tract of mouse embryos at E10.5, and distributed in the bladder at E15.5. Expression of ISL1 in zebrafish larvae, staged 48 hpf, was detected in a small region of the developing pronephros, supporting the observations in mice. These genetic and developmental biology data support *ISL1* as a major susceptibility gene for CBE, and as a regulator of urinary tract development [5]. ISL1 is a member of LIM/Homeodomain (LHX) family of transcription factor genes, located at human chromosome 5, and has been shown to interact with estrogen receptor alpha [6]. 

Here, we aimed to determine the molecular and functional consequences of *ISL1* variants and estimate the dependence of ISL1 protein on other predicted candidates. We used: (i) computational analysis of conserved sequence motifs based on a Bayesian algorithm, and (ii) 3D structural modeling. Furthermore, we looked into long non-coding RNAs (lncRNAs) residing within the *ISL1* region, aiming to predict their targets.

## 2. Materials and Methods 

The ISL1 sequence variants (rs9291768, rs6874700, and c.137C > G (p.Ala46Gly), known to be associated with CBE, were selected for the comprehensive analysis. From our own previous studies, we chose the variants rs9291768 and rs6874700 for further analysis, as these were the variants with the most significant association with classic bladder exstrophy. Although the coding variants rs2303751 and rs41268419 were previously found to be in linkage-disequilibrium with rs9291768 and rs6874700, we did not follow up on these two coding variants in the current analysis, since these variants are synonymous in nature and do not alter the protein structure, nor function. Variant c.137C > G was chosen because it was found to be associated with BEEC in an independent study by Arkani et al. 2018. The minor allele frequencies of the investigated variants have been provided, according to genomAD database [7] in Supplementary File 8.

### 2.1. Sequence Homology-Based Single Nucleotide Polymorphism Prediction Using PolyPhen-2 & CADD 

Pathogenicity of the ISL1 p.Ala46Gly variant (corresponding to c.137C > G) was ascertained using PolyPhen-2 (Polymorphism Phenotyping-2) [8]. The methodology is based on the procedure used previously [9] with some modifications. For PolyPhen-2, a non-synonymous single nucleotide polymorphism (SNP), present in the coding region of a gene, is predicted to be “damaging” if the prediction score is above the threshold value (cutoff is 0.96). For SNP classification using CADD [10], the required variant information such as chromosome number, position, reference base pair, and altered base pair have been used as input files for predicting and generating CADD scores. 

### 2.2. Identification of Conserved Residues and Sequence Motifs Using Consurf

The UniProtKB amino acid sequence of the sixteen proteins in FASTA format, was used as input for computational analysis of conserved sequences and motifs using Consurf web server [11], carrying out evolutionary conservation analysis based on a Bayesian algorithm. The output of the Consurf analysis shows degrees of conservation of an amino acid residue in the test protein, by means of color coding (conservation scores: 1–4 variable, 5–6 intermediate, and 7–9 conserved). Exposed (functional) and buried (structural) residues, with high conservation levels, were scored in the amino acid sequence respectively.

### 2.3. Computational 3D Structural Modelling

3D structure models were built using I-TASSER (Iterative Threading ASSEmbly Refinement), which employs an integrated combinatorial approach comprising of comparative modelling, threading and ab initio modeling [12] using the procedure adopted by Dakal et al. [13]. The stereo chemical status of modelled structures was validated using PROCHECK at SAVES [14,15]. The superimposition of ISL1 Ala46Gly, and ISL1 wild type protein, was done using the Superpose version 1 program (wishart.biology.ualberta.ca/superpose), with a minimum sequence similarity of 80%, similarity and dissimilarity cutoffs of 2 and 3Å respectively, and subdomain matching “on”. The predicted ISL1 model was successfully obtained from I-TASSER using the best quality structure among generated models, derived by using the template (PDB ID: 4JCJA) with confidence score (Cscore:4.35), Template Modeling Score (TM:0.26 ± 0.08), and the Root-Mean-Square Deviation (RMSD:17.6 ± 2.6 Å.) for wild type protein. In the same way, for variant c.137C > G the best predicted model was obtained with Cscore: 4.54; TM: 0.24 ± 0.07; RMSD: 18.1 ± 2.4 Å. The sequence similarity with the template (PDB ID: 4JCJA) was 37% and model length was 359 aa for both structural models generated.

### 2.4. Prediction of Functional Consequences of Non-Coding Variants and Prediction of Long Noncoding RNA Targets 

In order to predict the functional consequences of non-coding variants (rs9291768, rs6874700), GWAVA was used. The dbSNP rsIDs of these variants were used as the input files, and prediction scores were retrieved based on annotations available from ENCODE/GENCODE [16]. Additionally, in the present study, lncRNA targets were predicted based on the prediction of their cis function [17,18,19]. The closest coding genes 10 kb upstream and downstream of lncRNAs (may not be its direct target), were screened using the BEDTools v2.25.0 program [20] Using the UCSC Genome browser, the genes located downstream of rs9291768 and rs6874700 were mapped. LncTar was used for predicting if the gene upstream or downstream of rs9291768 such as *ISL1* and *PARP8* are RNA targets of lncRNA [21], using the default parameters. 

### 2.5. Generation of Long Noncoding RNA Secondary Structures

To assess the impacts of SNPs on lncRNA secondary structures, we first extracted lncRNA transcript sequences from the human reference genome (hg38 version), according to the lncRNA transcript BED file, as Ref-transcripts. The secondary structure of the lncRNA was predicted using the RNAfold program [22], which constructs the lncRNA secondary structure using the input sequence, and by calculating the minimal free energy (MFE, ΔG). Energy change of RNA structures (ΔΔG) was calculated by the minimal free energy differences using ΔΔG = [ΔG_alt_ − ΔG_ref_], where ΔG_ref_ and ΔG_alt_ are the MFEs of the reference and altered transcript, respectively. The detailed information regarding all IDs (proteins, genes, lncRNA), tool versions and databases, has been provided in Supplementary File 5.

### 2.6. Protein-Protein Interaction Network Analysis

We analyzed 72 genes that, according to literature, are putatively associated with BEEC (Supplementary File 6), for functional protein–protein interactions (PPI) via STRING (Search tool for the Retrieval of Interacting Genes/Proteins) [23]. STRING is a large-scale database of experimentally verified and manually curated protein-protein interactions, as well as others that are inferred on the basis of shared signals across genomes, text mining, gene co-expression, and protein homology [24]. That means, edges in PPI networks that are derived from STRING, represent shared biological functions and not necessarily physical interactions. These genes were also investigated for their functionality in different developmental stages of mice, with regards to their bladder and cloacal formation using mouse gene expression database (GXD) [25]. 

## 3. Results

### 3.1. Characterization of Genetic Variants and Associated Long Noncoding RNA 

**Figure 1:**
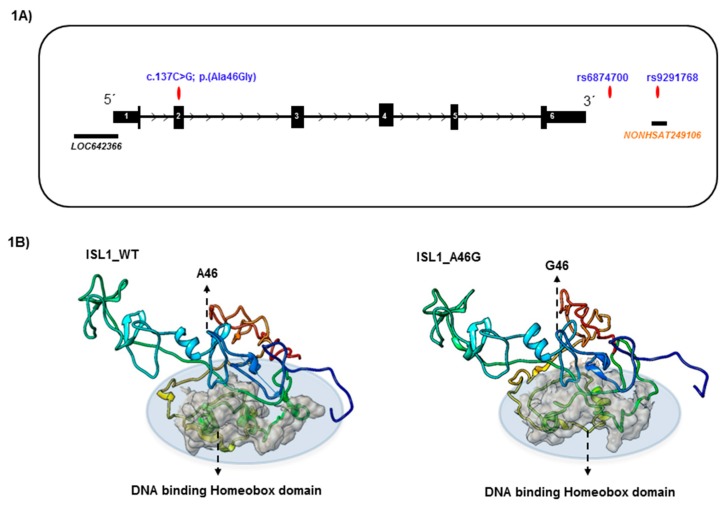
Characterization of *ISL1* genomic variants. (**A**) The exon/intron structure of *ISL1* with genomic variants is presented. (**B**) Structural modeling of ISL1 wild type and c.137C > G (p.Ala46Gly)) mutant protein.

All rsIDs associated with non-coding regions were subjected to GWAVA analysis. We found that all rsIDs showed a GWAVA score less than 0.5, which indicates that the variants are non-functional and they are likely to be associated with the disease conditions (Supplementary File 4). From the GWAVA analysis, we also computed the distance of the rsIDs associated SNPs from the nearest transcription start site (TSS). We found that the nearest transcript start sites were 4288 and 6467 bp rs6874700 and rs9291768 respectively. This also indicates that these non-coding variants are potentially disease-associated, as most disease-associated variants are generally found nearer TSSs [16]. The two *ISL1* associated markers, rs9291768 and rs6874700, were analyzed for their potential association with any regulatory function (Figure 1a). While our analysis did not reveal regulatory functions for rs6874700, rs9291768 was found to be associated (overlapped) with the region annotated as lncRNA (NONCODE_v5_lncRNA: NONHSAT249106), which was located 27.2 Kbp downstream of *ISL1* (Figure 1). In addition, apart from a few pseudogenes (*HMGB1P47*, RNU6-1296P, RNU6-480P, *RNA5SP182*, *RP11*), we identified one protein-coding gene, *PARP8* (536,870 bp upstream), in the vicinity of rs9291768. Since *ISL1* and *PARP8* are the only protein coding genes mapped closely to rs9291768, we considered the mRNA of these two genes for further analysis. Our analysis, using web-tool LncTar, confirmed that neither *ISL1* nor *PARP8* mRNA is a target of this lncRNA (NONCODE_v5_lncRNA: NONHSAT249106) and therefore, the potential functional consequence of SNP rs9291768 has been assumed to be neutral. However, we cannot exclude the possibility that lncRNA NONHSAT249106 may have a functional impact on the transcripts of distantly located genes. 

Considering the effect of SNP rs9291768 on the secondary structure of lncRNA (*NONHSAT249106*), which could potentially alter its stability, expression, or function, we compared the minimal free energy (MFE) secondary structure and the centroid secondary structure generated from the transcripts of both reference and altered IncRNAs, by using the RNAfold program. We found that the lncRNA secondary structure of both the reference and altered (re9291768) transcript showed a similar secondary structure, with MFE = −11.40Kcal/mol, suggesting that rs9291768 entails no structural change in lncRNA structure (Figure S1). Apart from this 3’lncRNA (*NONHSAT249106*), one other notable lncRNA, (LonLOC642366), overlaps with first exon of the *ISL1* gene, whose function is still unclear.

### 3.2. Structural Modeling of ISL1 Wild Type Protein and the ISL1 Variant, p.Ala46Gly

**Figure 2:**
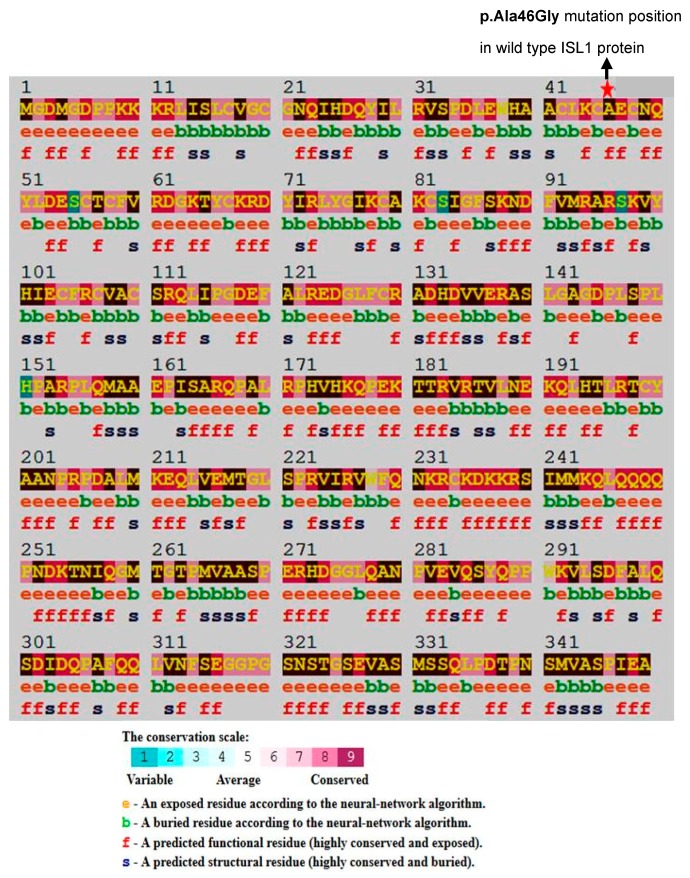
Structural conformation and conservation analysis of p.Ala46Gly. Analysis of evolutionary conserved amino acid residues of p.Ala46Gly by ConSurf. The color coding bar shows the coloring scheme representation of conservation scores.

A novel missense variant, c.137C > G (p.Ala46Gly), was investigated to build the three-dimensional structure of ISL1 wild type, and the p.Ala46Gly variant protein. The structures were generated using the wild type amino acid sequences (UniProt ID: P61371) and the mutant amino acid sequence (with p.Ala46Gly), by the Iterative Threading ASSEmbly Refinement method (I-TASSER) available online. For generating both models, I-TASSER used the template of the 4JCJ crystal structure from the PDB database [26]. Since alanine (wild type) and glycine (variant type) are both small and non-polar amino acids, we expected no extensive structural changes in the variant ISL1 Ala46Gly protein, compared to the wild type. Consistent with this, structural modeling of the ISL1 wild type and the variant protein showed no change in the local, as well as the overall, protein structure (Figure 1B, Supplementary File 3). We next subjected the amino acid of the ISL1 wild type protein to Consurf, which computes the evolutionarily conserved, structural (buried), and functional (exposed) residues, in a protein. We found that the p.Ala46Gly position is highly conserved, which suggests that this variant might be functional in nature (Figure 2, Supplementary File 7). Additionally, structure homology based SNP prediction tools such as PolyPhen-2 and CADD also predicted the ISL1 p.Ala46Gly variant protein to be damaging (score = 0.997; sensitivity: 0.41; specificity: 0.98; and CADD of score= 25.8). The stereochemical quality of the modeled structures were validated for steric clashes, using a Ramachandran plot analysis by PROCHECK, available at SAVES. The ISL1 wild type and ISL1 Ala46Gly protein variants modelled using I-TASSER, possess approximately 95% of residue accurately, while the remaining 5.6% in the wild type, and 3.3% in Ala46Gly variant protein, were found in disallowed regions within the Ramachandran plot. To explore the position of the substitution in the reconstructed protein structure, we have computed the variant c.137C > G in dG, using I-Mutant version 2, comparing it to the *ISL1* wild type, and observed that the *ISL1* c.137C > G resulted in decreased protein stability.

We assume that the C>G variant in *ISL1* at position c.137 might have functional consequences. Being a transcription factor, ISL1 protein acts by binding to the promoter of the target genes, and thereby, modulates their gene expression. From a structural point of view, the p.Ala46Gly is present at a loop turn, close to the N-terminal region of the ISL1 protein, and is also close to the DNA binding homeobox domain (amino acids 181–240). Since the LIM domain of ISL1 is known to interact with estrogen receptor α, the p.Ala46Gly variant protein may influence ISL1-estrogen receptor α interactions.

### 3.3. Protein–Protein InteractionsNetwork Analysis 

**Figure 3:**
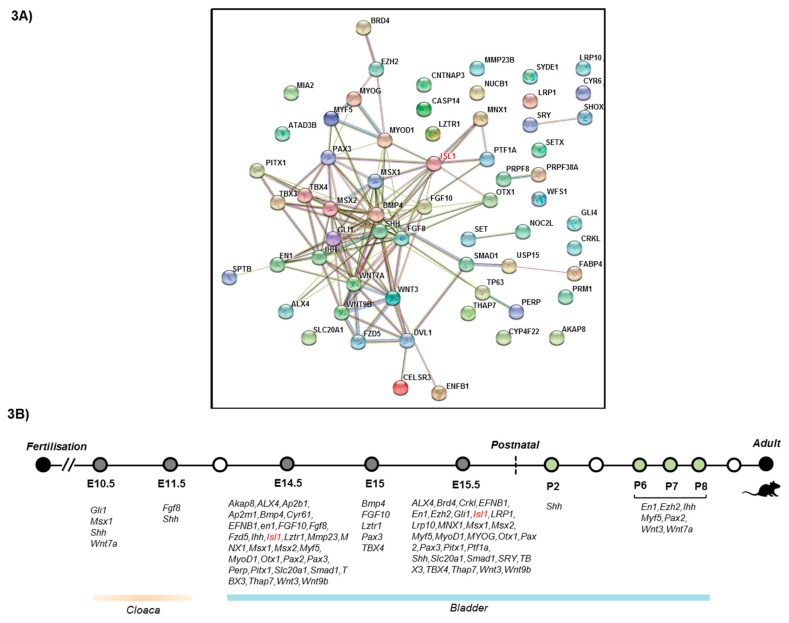
STRING analysis showing enrichment of protein-protein interactions. (**A**) Protein-protein interactions of 72 genes linked to the bladder exstrophy–epispadias complex (BEEC) are shown. ISL1, as a major interaction partner, is marked in red. (**B**) Distributions of essential and viable genes for the formation of the urinary bladder and cloaca across different stages of mouse development.

Functional PPI analysis via STRING resulted into a network of 50 proteins (Figure 3A). This was done to understand potential functional associations of ISL1 with other proteins. Notably, members of the pre-mRNA processing factors (PRPF8, PRPF38), and proteins associated with sex chromosomes (SRY, SHOX), were highly connected with each member without any additional interacting partners. The network showed a high statistical overrepresentation (hyper-geometric test, false discovery rate <1%) of genes involved into several Kyoto Encyclopedia of Genes and Genomes (KEGG) pathways [27], namely Hedgehog, Hippo, and Wnt signaling, which is consistent with the agreement with the previous investigations [28,29,30]. Notably, our approach does not predict the chronological contribution of these proteins to the disease phenotype. But, our analysis suggests that mutational events in the *ISL1* gene alone are not sufficient enough to create a severe phenotype. 

To better understand the functionality of these selective 50 genes in a STRING network, we investigated their putative involvement in different developmental stages of mice (Figure 3B). We found that all these genes are distributed from embryonic to postnatal stages (E14.5-P8), in the urinary bladder and in early embryonic stages (E10.5–E11.5), for the cloacal region. We also noticed that the expression patterns of certain genes (e.g., PAX3, TBX3) are required during all early stages of bladder formation, while interplay with several other genes related to the Wnt signaling pathway are required as development proceeds.

## 4. Discussion

In an attempt to strengthen our understanding of the involvement of the *ISL1* gene in the etiology of the BEEC, our analysis provides novel insights about the consequences of genetic variants embedded within the *ISL1* genomic landscape. In the present study, we investigated four genomic variants, and one recently introduced missense variant, linked to the BEEC. By performing structural analysis, we showed that although rs9291768, which is a part of lncRNA (NONCODE_v5_lncRNA: NONHSAT249106), *ISL1* mRNA does not seem to be the main target of this lncRNA. Our amino acid sequence conservation analysis using Consurf, demonstrates that the p.Ala46Gly position (in LIM domain of ISL1 gene) is evolutionary conserved. However, the homeobox domain (amino acids 181-240), at the C-terminal region of ISL1, plays a unique role in DNA binding and the amino acid change of p.Ala46Gly, which resides at a loop turn close to the N-terminal region. Thus, it might have functional effects possibly interfering with the binding of ISL1 to estrogen receptor α. 

ISL1 protein has been very strongly conserved during evolution, perhaps due to the involvement of different protein interaction networks. The multiplicity of ISL1 function is mirrored by its role in different diseases, such as BEEC, diabetes type I/II, and congenital heart defects [31,32,33]. The dependence of all these functions stemming from one and the same molecular structure, might in part be responsible for the paucity of disease-specific substitutions.

To understand the functional role of the ISL1 protein and other mutually exclusive proteins, for their involvement in the formation of the BEEC in more depth, we selected 72 genes which have been putatively linked to the BEEC, and performed PPI analysis. We found an interaction network between 50 proteins previously associated with the BEEC. Our approach does not predict the chronological contribution of these proteins to the disease phenotype. As the ISL1 protein shows up as a major interaction partner in our network analysis, we speculate that the involvement of mutational events in the *ISL1* gene alone is not sufficient enough to create a severe phenotype. In addition, pathway enrichment analysis further suggests an involvement of hedgehog, hippo, and Wnt signaling pathways, in the etiology of BEEC. This in turn suggests that regardless of the complexity of the BEEC, pathway crosstalk also plays a significant role in pathogenesis of the disease. Importantly, the molecular framework of pathway integration, with regard to the ISL1 protein, needs further investigation.

In conclusion, our study characterized three genetic variants within the coding and non-coding region of *ISL1*. Previously, GWAS has identified the *ISL1* locus to be associated with BEEC. Here, we investigated whether the most significantly associated variants rs9291768 and rs6874700 might constitute potential targets of the lncRNA (NONHSAT249106), residing in their close vicinity. However, the results of our analysis do not support this hypothesis. Interestingly, our analysis suggests that the *ISL1* variant c.137C > G, results in decreased protein stability. In addition, by integrating multiple parameters, we provide novel insights about the involvement of *ISL1* in the etiology of BEEC.

## Supplementary Materials

The following are available online at http://www.mdpi.com/2073-4425/9/12/609/s1, Figure S1: Long non coding RNA overlapping with rs9291768 (upper) and secondary structure of Long noncoding RNA (NONCODE_v5_lncRNA: NONHSAT249106) associated with rs9291768 (A: Secondary Structure MFE = −11.40 kcal/mol, B: Centroid Secondary Structure MFE = −10.30Kcal/mol) is shown (below), Figure S2: Genomic landscape of rs6874700, Supplementary File 3: (ISL1 local protein changes), Supplementary File 4: (GWAVA and Ramachandran plots), Supplementary File 5: (Information of IDs, tool versions and databases), Supplementary File 6: (List of genes involved in BEEC), Supplementary File 7: (ISL1 alignment), Supplementary File 8: (Minor allele frequencies of the investigated variants).
