# Supplementary material for: Towards a Central Role of ISL1 in the Bladder Exstrophy–Epispadias Complex (BEEC): Computational Characterization of Genetic Variants and Structural Modelling
Source: Genes (Basel). 2018 Dec 5;9(12):609. doi: 10.3390/genes9120609 (PMC6315746; doi:10.3390/genes9120609)
Supplement: Supplementary file 1 [file genes-09-00609-s001.zip › genes-405097-final-suppl/Revision Supplementary File 1 and 2.docx]

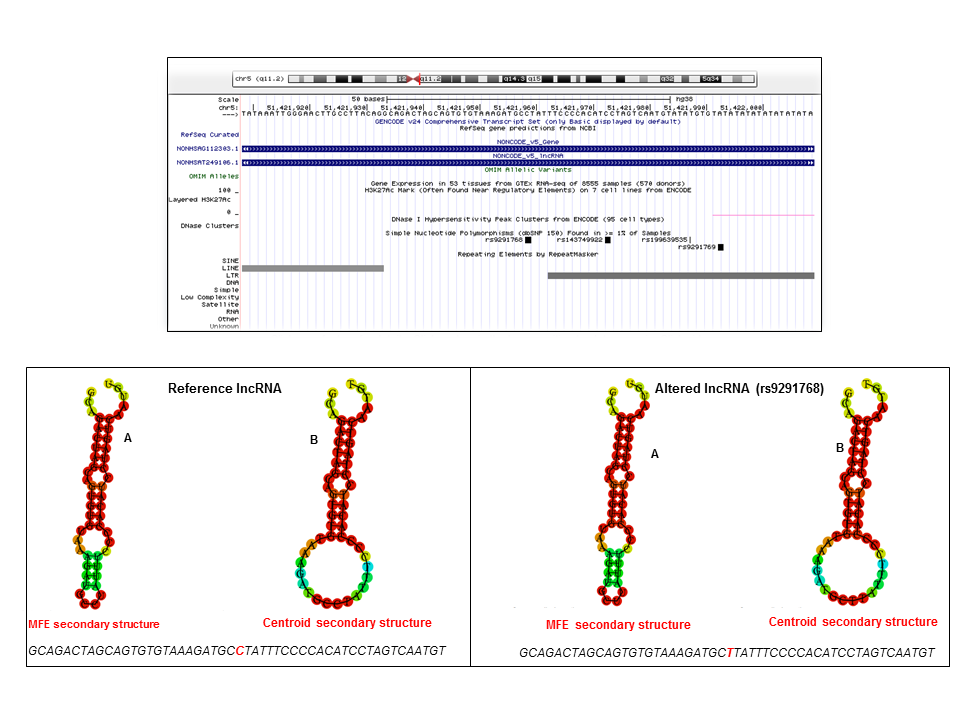


**Figure S1:** Long non coding RNA overlapping with rs9291768 (upper) and secondary structure of Long noncoding RNA (NONCODE_v5_lncRNA: NONHSAT249106) associated with rs9291768 (A: Secondary Structure MFE=-11.40 kcal/mol, B: Centroid Secondary Structure MFE= -10.30Kcal/mol) is shown (below)


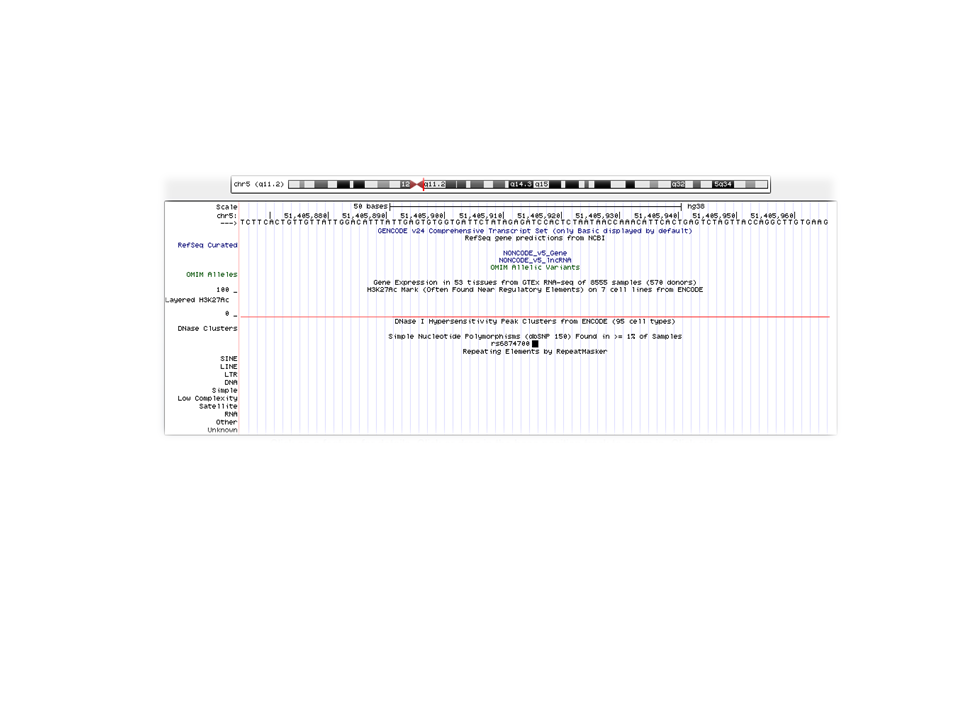


**Figure S2:** Genomic 307 landscape of rs6874700.
