# Supplementary material for: Towards a Central Role of ISL1 in the Bladder Exstrophy–Epispadias Complex (BEEC): Computational Characterization of Genetic Variants and Structural Modelling
Source: Genes (Basel). 2018 Dec 5;9(12):609. doi: 10.3390/genes9120609 (PMC6315746; doi:10.3390/genes9120609)
Supplement: Supplementary file 1 [file genes-09-00609-s001.zip › genes-405097-final-suppl/Revision Supplementary File 3 local protein changes.pptx]

## Slide 1
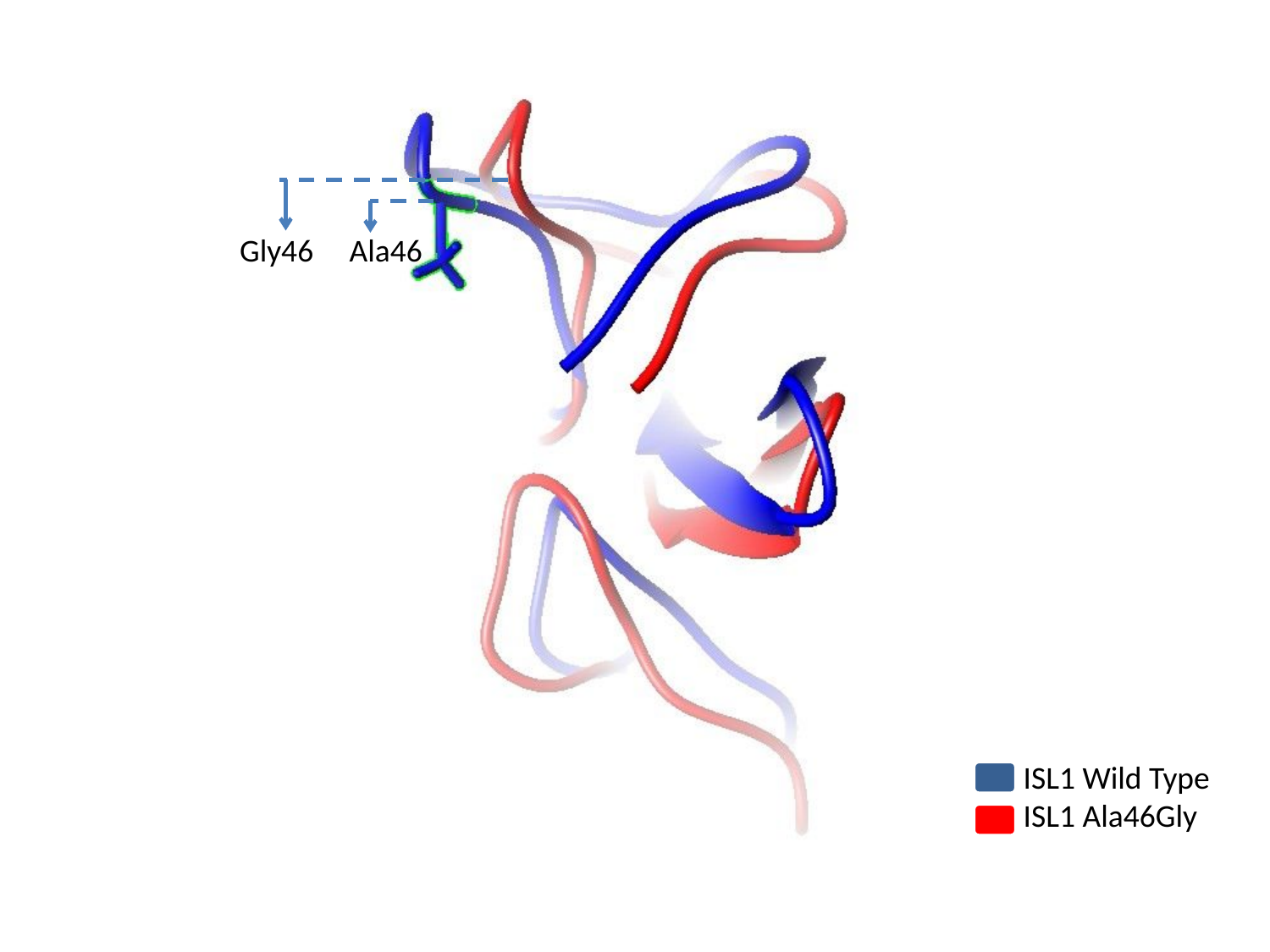

Gly46 Ala46
ISL1 Wild Type
ISL1 Ala46Gly

## Slide 2
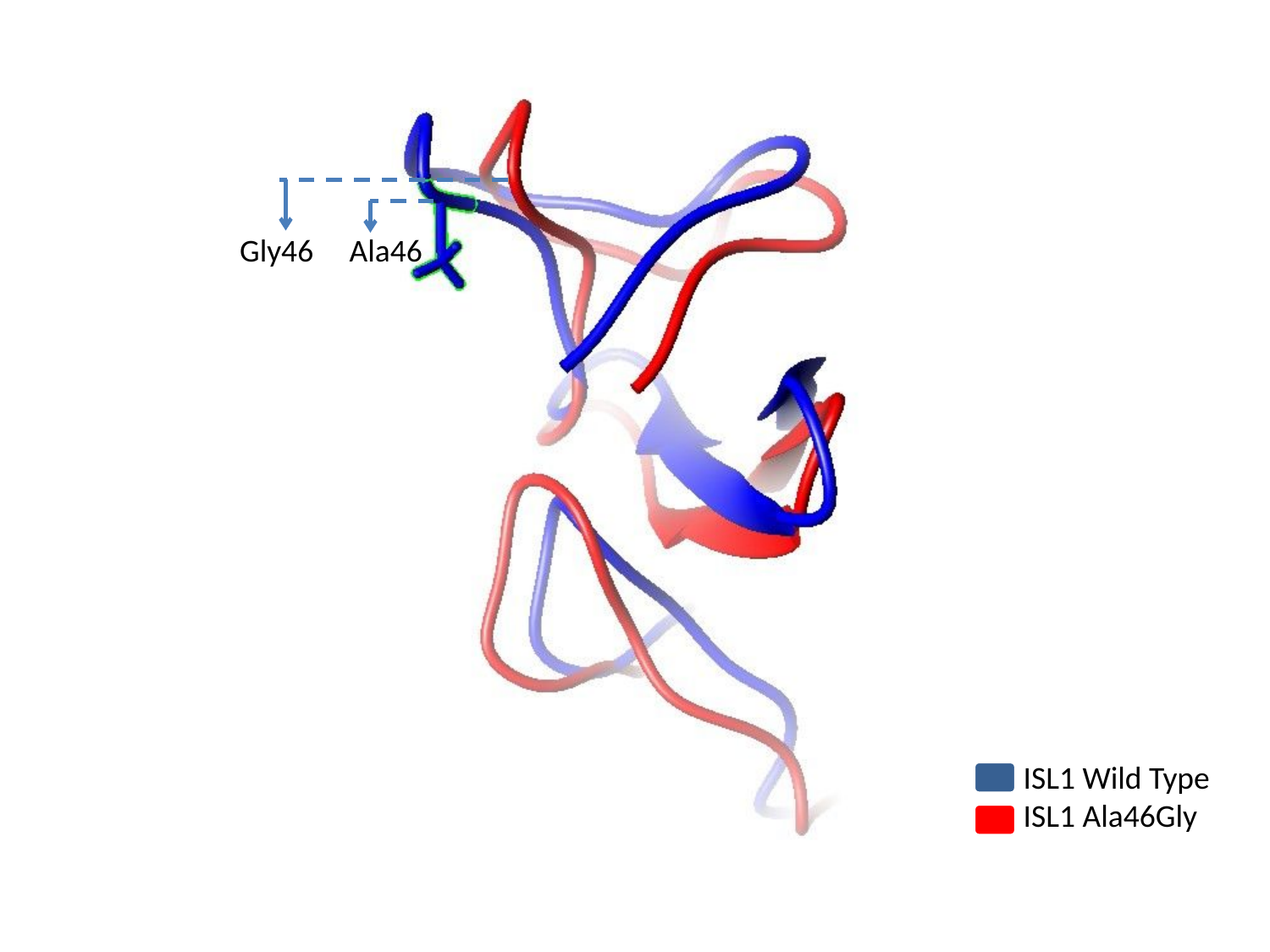

Gly46 Ala46
ISL1 Wild Type
ISL1 Ala46Gly

## Slide 3
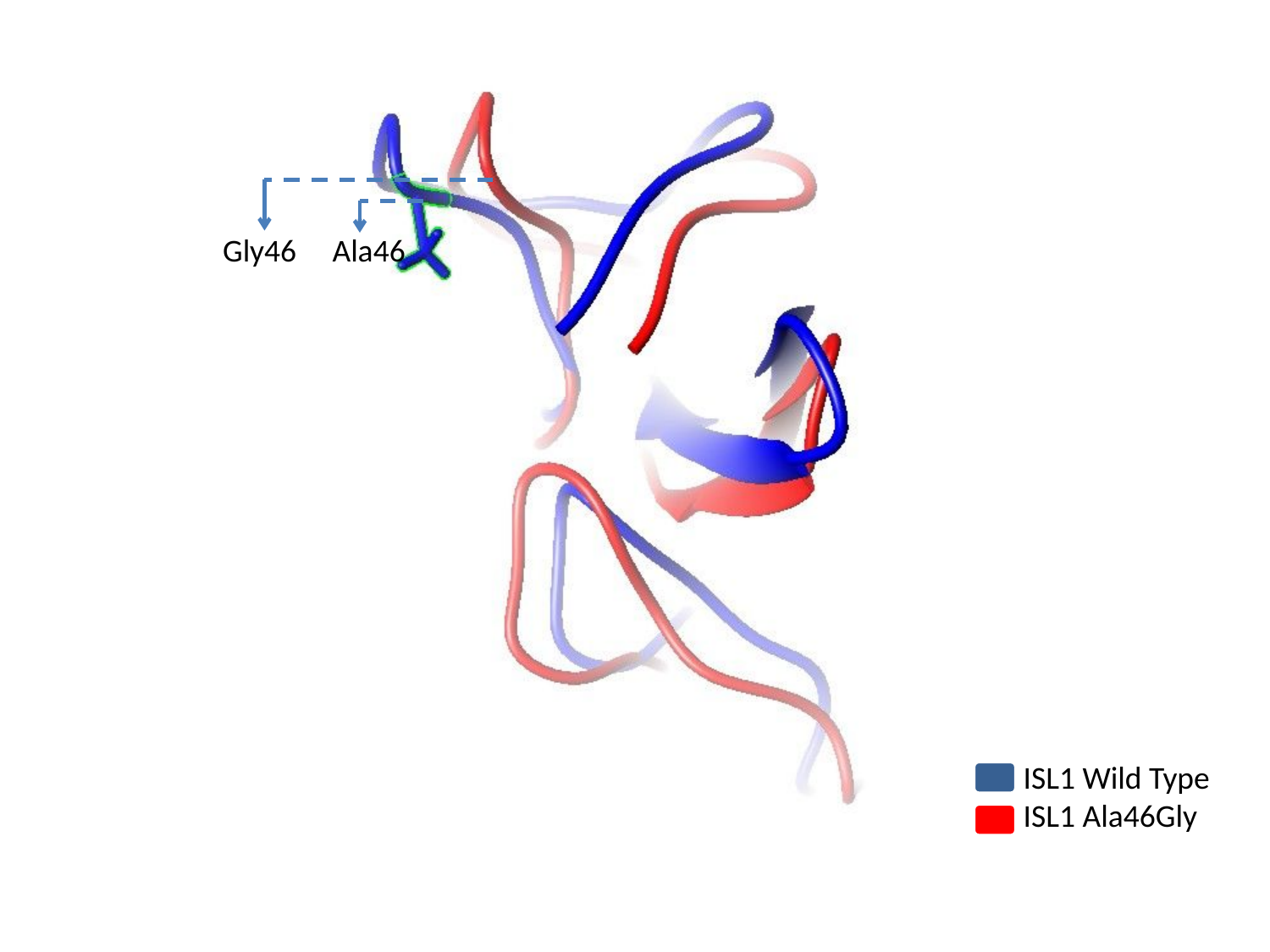

Gly46 Ala46
ISL1 Wild Type
ISL1 Ala46Gly

## Slide 4
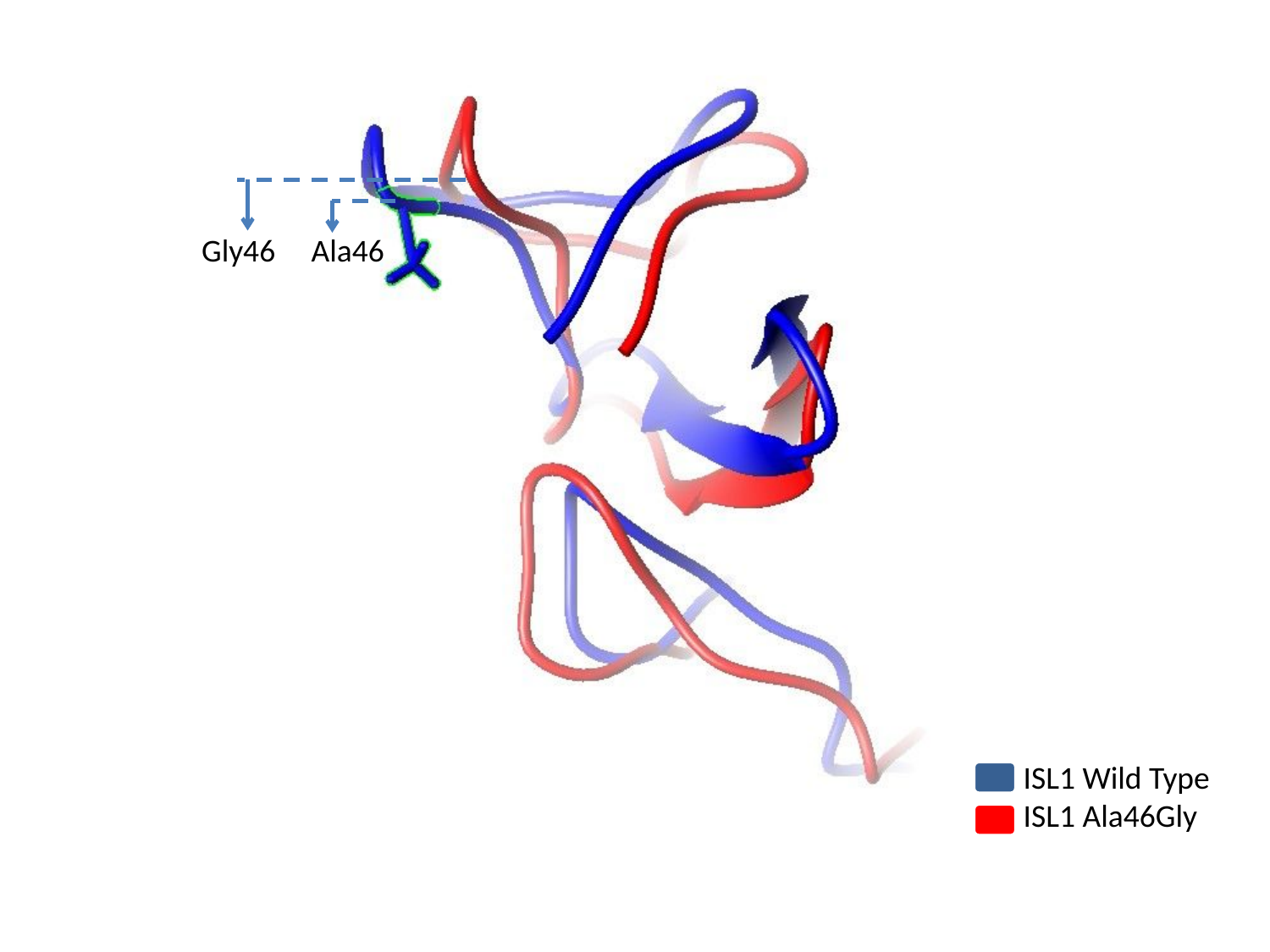

Gly46 Ala46
ISL1 Wild Type
ISL1 Ala46Gly
