# Supplementary material for: Towards a Central Role of ISL1 in the Bladder Exstrophy–Epispadias Complex (BEEC): Computational Characterization of Genetic Variants and Structural Modelling
Source: Genes (Basel). 2018 Dec 5;9(12):609. doi: 10.3390/genes9120609 (PMC6315746; doi:10.3390/genes9120609)
Supplement: Supplementary file 1 [file genes-09-00609-s001.zip › genes-405097-final-suppl/Revision Supplementary File 5 weblinks.docx]

| S.No. | Analyses | Protein /LncRNA | Gene | Protocol/  Methodology | webserver |
| --- | --- | --- | --- | --- | --- |
| 1 | For SNP classification-PolyPhen-2 | ISL1 wild type  ISL1 Ala46Gly | *ISL1* | The chromosome no., position, reference base pair and altered base pair was used as input file | http://genetics.bwh.harvard.edu/pph2/ |
| 2 | For SNP classification-CADD score | ISL1 wild type  ISL1 Ala46Gly | *ISL1* | The chromosome no., position, reference base pair and altered base pair was used as input file | https://cadd.gs.washington.edu/snv |
| 3 | Conservation analysis | ISL1 wild type | *ISL1* | The MSA file created using CLUSTAL was used as input file | consurf.tau.ac.il |
| 4 | Protein structure modeling | ISL1 wild type  ISL1 Ala46Gly |  | I-Tasser, which computes tertiary structure using an integrated method comprising MSA, threading and ab-initio modeling | https://zhanglab.ccmb.med.umich.edu/I-TASSER/ |
| 5 | Structure validation | ISL1 wild type  ISL1 Ala46Gly |  | Default setting | http://servicesn.mbi.ucla.edu/PROCHECK/ |
| 6 | Structure superimposition | ISL1 Ala46Gly onto the ISL1 wild type |  | Minimum sequence similarity 80%  Similiarity and dissimilarity cutoff of 2 and 3Å, respectively  Subdomain matching “on”. | wishart.biology.ualberta.ca/superpose |
| 7 | Computation of Free Energy Change Value (ddG) and stability of mutant protein | ISL1 Ala46Gly wrt  ISL1 wild type | *ISL1* | The ddG was calculated for ISL1 Ala46Gly wrt ISL1wild type at temp 27 degree and pH 7.0 using i-Mutant ver 2. | http://folding.biofold.org/cgi-bin/i-mutant2.0.cgi |
| 8 | LncRNA structure prediction | NONHSAT249106  (NONCODE_v5_lncRNA) | *--* | The lncRNA transcript sequences from human reference genome (hg38 version)  Was used as an input file. | RNAfold program |
