# Supplementary material for: Towards a Central Role of ISL1 in the Bladder Exstrophy–Epispadias Complex (BEEC): Computational Characterization of Genetic Variants and Structural Modelling
Source: Genes (Basel). 2018 Dec 5;9(12):609. doi: 10.3390/genes9120609 (PMC6315746; doi:10.3390/genes9120609)
Supplement: Supplementary file 1 [file genes-09-00609-s001.zip › genes-405097-final-suppl/Revision Supplementary File 6 72 gene list.docx]

Supplementary Table 1: List of genes selected in the study

| *AKAP8* ^57^ | *FGF10* ^48^ | *MYF5* ^39^ | *SHH* ^51^ |
| --- | --- | --- | --- |
| *ALX4* ^50^ | *FGF8* ^51^ | *MYOD* ^39^ | *SHOX* ^58^ |
| *AP2* ^39^ | *FZD5* ^37^ | *MYOGENIN* ^39^ | *SLC20A1* ^57^ |
| *ATAD3B* ^62^ | *GLi1* ^50^ | *NOC2L* ^41^ | *SMAD1* ^44^ |
| *BMP4* ^45^ | *GLi3* ^50^ | *NUCB1* ^57^ | *SMAD4* ^64^ |
| *BMPRLA* ^51^ | *GLi4* ^46^ | *OTX1* ^47^ | *SPTB* ^57^ |
| *BRD4* ^43^ | *HLXB9* ^59^ | *P63* ^40^ | *SRY* ^52^ |
| *CASP14* ^43^ | *IHH* ^61^ | *PAX2A* ^44^ | *SYDE1* ^43^ |
| *CELSR3* ^57^ | *ISL1* ^42^ | *PAX3* ^60^ | *TBX3* ^50^ |
| *CNTNAP3* ^38^ | *LRP1* ^37^ | *PERP* ^49^ | *TBX4* ^50^ |
| *CRKL* ^54^ | *LRP10* ^37^ | *PITX1* ^50^ | *TGF* ^44^ |
| *CYP4F22* ^43^ | *LZTR1* ^54^ | *PRM1* ^63^ | *THAP7* ^54^ |
| *CYR61* ^43^ | *MAB2112* ^50^ | *PRPF38A* ^57^ | *WFS1* ^57^ |
| *DELTANP63* ^40^ | *MIA2* ^57^ | *PRPF8* ^57^ | *WITZ* ^43^ |
| *DVL1* ^41^ | *MMP23B* ^41^ | *PTCH1* ^44^ | *WNT3 ^56^* |
| *EFNB1* ^62^ | *MNX1* ^59^ | *Ptf1a* ^59^ | *WNT7A* ^37^ |
| *EN1* ^35^ | *MSX1* ^53^ | *SET* ^65^ | *WNT9B* ^56^ |
| *EZH2* ^36^ | *MSX2* ^53^ | *SETX* ^57^ |  |

References:

1. Ahn, K.; Mishina, Y.; Hanks, M.C.; Behringer, R.R.; Crenshaw, E.B., 3rd. BMPR-IA signaling is required for the formation of the apical ectodermal ridge and dorsal-ventral patterning of the limb. Development 2001, 128, 4449-4461.
2. San, B.; Chrispijn, N.D.; Wittkopp, N.; van Heeringen, S.J.; Lagendijk, A.K.; Aben, M.; Bakkers, J.; Ketting, R.F.; Kamminga, L.M. Normal formation of a vertebrate body plan and loss of tissue maintenance in the absence of ezh2. Sci Rep 2016, 6, 24658, doi:10.1038/srep24658.
3. Baranowska Korberg, I.; Hofmeister, W.; Markljung, E.; Cao, J.; Nilsson, D.; Ludwig, M.; Draaken, M.; Holmdahl, G.; Barker, G.; Reutter, H., et al. WNT3 involvement in human bladder exstrophy and cloaca development in zebrafish. Hum Mol Genet 2015, 24, 5069-5078, doi:10.1093/hmg/ddv225.
4. Boyadjiev, S.A.; South, S.T.; Radford, C.L.; Patel, A.; Zhang, G.; Hur, D.J.; Thomas, G.H.; Gearhart, J.P.; Stetten, G. A reciprocal translocation 46,XY,t(8;9)(p11.2;q13) in a bladder exstrophy patient disrupts CNTNAP3 and presents evidence of a pericentromeric duplication on chromosome 9. Genomics 2005, 85, 622-629, doi:10.1016/j.ygeno.2005.01.002.
5. Brewer, S.; Williams, T. Loss of AP-2alpha impacts multiple aspects of ventral body wall development and closure. Dev Biol 2004, 267, 399-417, doi:10.1016/j.ydbio.2003.11.021.
6. Cheng, W.; Jacobs, W.B.; Zhang, J.J.; Moro, A.; Park, J.H.; Kushida, M.; Qiu, W.; Mills, A.A.; Kim, P.C. DeltaNp63 plays an anti-apoptotic role in ventral bladder development. Development 2006, 133, 4783-4792, doi:10.1242/dev.02621.
7. Collu, M.; Yuksel, S.; Sirin, B.K.; Abbasoglu, L.; Alanay, Y. Is 1p36 deletion associated with anterior body wall defects? Am J Med Genet A 2016, 170, 1889-1894, doi:10.1002/ajmg.a.37666.
8. Draaken, M.; Knapp, M.; Pennimpede, T.; Schmidt, J.M.; Ebert, A.K.; Rosch, W.; Stein, R.; Utsch, B.; Hirsch, K.; Boemers, T.M., et al. Genome-wide association study and meta-analysis identify ISL1 as genome-wide significant susceptibility gene for bladder exstrophy. PLoS Genet 2015, 11, e1005024, doi:10.1371/journal.pgen.1005024.
9. Draaken, M.; Proske, J.; Schramm, C.; Wittler, L.; Bartels, E.; Nothen, M.M.; Reutter, H.; Ludwig, M. Embryonic expression of the cysteine rich protein 61 (CYR61) gene: A candidate for the development of human epispadias. Birth Defects Res A Clin Mol Teratol 2010, 88, 546-550, doi:10.1002/bdra.20668.
10. Rasouly, H.M.; Lu, W. Lower urinary tract development and disease. Wiley Interdiscip Rev Syst Biol Med 2013, 5, 307-342, doi:10.1002/wsbm.1212.
11. Haraguchi, R.; Matsumaru, D.; Nakagata, N.; Miyagawa, S.; Suzuki, K.; Kitazawa, S.; Yamada, G. The hedgehog signal induced modulation of bone morphogenetic protein signaling: an essential signaling relay for urinary tract morphogenesis. PLoS One 2012, 7, e42245, doi:10.1371/journal.pone.0042245.
12. Hilger, A.C.; Halbritter, J.; Pennimpede, T.; van der Ven, A.; Sarma, G.; Braun, D.A.; Porath, J.D.; Kohl, S.; Hwang, D.Y.; Dworschak, G.C., et al. Targeted Resequencing of 29 Candidate Genes and Mouse Expression Studies Implicate ZIC3 and FOXF1 in Human VATER/VACTERL Association. Hum Mutat 2015, 36, 1150-1154, doi:10.1002/humu.22859.
13. Jorgez, C.J.; Rosenfeld, J.A.; Wilken, N.R.; Vangapandu, H.V.; Sahin, A.; Pham, D.; Carvalho, C.M.; Bandholz, A.; Miller, A.; Weaver, D.D., et al. Genitourinary defects associated with genomic deletions in 2p15 encompassing OTX1. PLoS One 2014, 9, e107028, doi:10.1371/journal.pone.0107028.
14. Kruger, V.; Khoshvaghti, M.; Reutter, H.; Vogt, H.; Boemers, T.M.; Ludwig, M. Investigation of FGF10 as a candidate gene in patients with anorectal malformations and exstrophy of the cloaca. Pediatr Surg Int 2008, 24, 893-897, doi:10.1007/s00383-008-2193-x.
15. Mahfuz, I.; Darling, T.; Wilkins, S.; White, S.; Cheng, W. New insights into the pathogenesis of bladder exstrophy-epispadias complex. J Pediatr Urol 2013, 9, 996-1005, doi:10.1016/j.jpurol.2013.05.001.
16. Matsumaru, D.; Haraguchi, R.; Moon, A.M.; Satoh, Y.; Nakagata, N.; Yamamura, K.; Takahashi, N.; Kitazawa, S.; Yamada, G. Genetic analysis of the role of Alx4 in the coordination of lower body and external genitalia formation. Eur J Hum Genet 2014, 22, 350-357, doi:10.1038/ejhg.2013.160.
17. Miyagawa, S.; Moon, A.; Haraguchi, R.; Inoue, C.; Harada, M.; Nakahara, C.; Suzuki, K.; Matsumaru, D.; Kaneko, T.; Matsuo, I., et al. Dosage-dependent hedgehog signals integrated with Wnt/beta-catenin signaling regulate external genitalia formation as an appendicular program. Development 2009, 136, 3969-3978, doi:10.1242/dev.039438.
18. Nishi, M.Y.; Martins, T.C.; Costa, E.M.; Mendonca, B.B.; Giron, A.M.; Domenice, S. Y chromosome aberration in a patient with cloacal-bladder exstrophy-epispadias complex: an unusual finding. Arq Bras Endocrinol Metabol 2013, 57, 148-152.
19. Ogi, H.; Suzuki, K.; Ogino, Y.; Kamimura, M.; Miyado, M.; Ying, X.; Zhang, Z.; Shinohara, M.; Chen, Y.; Yamada, G. Ventral abdominal wall dysmorphogenesis of Msx1/Msx2 double-mutant mice. Anat Rec A Discov Mol Cell Evol Biol 2005, 284, 424-430, doi:10.1002/ar.a.20180.
20. Pierquin G., Uwineza A. 22q11.2 microduplication in a patient with bladder exstrophy and delayed psychomotor development. Eur. J. Hum. Genet. 2012;20(Suppl. 1):89. [abstr].
21. Reutter, H.; Draaken, M.; Pennimpede, T.; Wittler, L.; Brockschmidt, F.F.; Ebert, A.K.; Bartels, E.; Rosch, W.; Boemers, T.M.; Hirsch, K., et al. Genome-wide association study and mouse expression data identify a highly conserved 32 kb intergenic region between WNT3 and WNT9b as possible susceptibility locus for isolated classic exstrophy of the bladder. Hum Mol Genet 2014, 23, 5536-5544, doi:10.1093/hmg/ddu259.
22. Reutter, H.; Keppler-Noreuil, K.; C, E.K.; Thiele, H.; Yamada, G.; Ludwig, M. Genetics of Bladder-Exstrophy-Epispadias Complex (BEEC): Systematic Elucidation of Mendelian and Multifactorial Phenotypes. Curr Genomics 2016, 17, 4-13, doi:10.2174/1389202916666151014221806.
23. Soderhall, C.; Lundin, J.; Lagerstedt-Robinson, K.; Grigelioniene, G.; Lackgren, G.; Kockum, C.C.; Nordenskjold, A. A case with bladder exstrophy and unbalanced X chromosome rearrangement. Eur J Pediatr Surg 2014, 24, 353-359, doi:10.1055/s-0033-1349056.
24. Thompson, N.; Gesina, E.; Scheinert, P.; Bucher, P.; Grapin-Botton, A. RNA profiling and chromatin immunoprecipitation-sequencing reveal that PTF1a stabilizes pancreas progenitor identity via the control of MNX1/HLXB9 and a network of other transcription factors. Mol Cell Biol 2012, 32, 1189-1199, doi:10.1128/mcb.06318-11.
25. Tremblay, P.; Dietrich, S.; Mericskay, M.; Schubert, F.R.; Li, Z.; Paulin, D. A crucial role for Pax3 in the development of the hypaxial musculature and the long-range migration of muscle precursors. Dev Biol 1998, 203, 49-61, doi:10.1006/dbio.1998.9041.
26. Vlangos, C.N.; Siuniak, A.; Ackley, T.; van Bokhoven, H.; Veltman, J.; Iyer, R.; Park, J.M.; Keppler-Noreuil, K.; Keegan, C.E. Comprehensive genetic analysis of OEIS complex reveals no evidence for a recurrent microdeletion or duplication. Am J Med Genet A 2011, 155A, 38-49, doi:10.1002/ajmg.a.33757.
27. von Lowtzow, C.; Hofmann, A.; Zhang, R.; Marsch, F.; Ebert, A.K.; Rosch, W.; Stein, R.; Boemers, T.M.; Hirsch, K.; Marcelis, C., et al. CNV analysis in 169 patients with bladder exstrophy-epispadias complex. BMC Med Genet 2016, 17, 35, doi:10.1186/s12881-016-0299-x.
28. Wittler, L.; Hilger, A.; Proske, J.; Pennimpede, T.; Draaken, M.; Ebert, A.K.; Rosch, W.; Stein, R.; Nothen, M.M.; Reutter, H., et al. Murine expression and mutation analyses of the prostate androgen-regulated mucin-like protein 1 (Parm1) gene, a candidate for human epispadias. Gene 2012, 506, 392-395, doi:10.1016/j.gene.2012.06.082.
29. Zhang, R.; Knapp, M.; Suzuki, K.; Kajioka, D.; Schmidt, J.M.; Winkler, J.; Yilmaz, O.; Pleschka, M.; Cao, J.; Kockum, C.C., et al. ISL1 is a major susceptibility gene for classic bladder exstrophy and a regulator of urinary tract development. Sci Rep 2017, 7, 42170, doi:10.1038/srep42170.
30. Reutter, H.; Thauvin-Robinet, C.; Boemers, T.M.; Rosch, W.H.; Ludwig, M. Bladder exstrophy-epispadias complex: Investigation of suppressor of variegation, enhancer of zeste and Trithorax (SET) as a candidate gene in a large cohort of patients. Scand J Urol Nephrol 2006, 40, 221-224, doi:10.1080/00365590600621204.
